# Mutant *EXT1* in Taiwanese Patients with Multiple Hereditary Exostoses

**DOI:** 10.7603/s40681-014-0011-4

**Published:** 2014-08-01

**Authors:** Wei-De Lin, Wuh-Liang Hwu, Chung-Hsing Wang, Fuu-Jen Tsai

**Affiliations:** 1 Department of Medical Research, China Medical University Hospital, Taichung, Taiwan; 2 Department of Pediatrics, China Medical University Hospital, Taichung, Taiwan; 3 Department of Medical Genetics, China Medical University Hospital, Taichung, Taiwan; 4 School of Post Baccalaureate Chinese Medicine, China Medical University, Taichung, Taiwan; 5 School of Medicine, China Medical University, Taichung, Taiwan; 6 School of Chinese Medicine, China Medical University, Taichung, Taiwan; 7 Department of Pediatrics and Medical Genetics, National Taiwan University Hospital and National Taiwan University School of Medicine, Taipei, Taiwan; 8 Department of Health and Nutrition Biotechnology, Asia University, Taichung, Taiwan; 9 Department of Pediatrics and Medical Genetics, China Medical University Hospital, No. 2, Yuh Der Road, 404 Taichung, Taiwan

**Keywords:** Multiple hereditary exostoses, EXT1, EXT2, Gene mutation

## Abstract

**Background:**

Multiple hereditary exostoses (MHE) is characterized by multiple benign projections of bone capped by cartilage, most numerous in metaphyses of long bones. HME are usually inherited in autosomal dominant mode, chief genes *EXT1* and *EXT2*.

**Methods:**

Two MHE patients were identified from clinic and enrolled in genetic study, complete coding regions of *EXT1* and *EXT2*, including intron/exon boundaries, sequenced via DNA samples drawn from participants.

**Results:**

DNA sequencing revealed mutant *EXT1* gene in both cases, within which frame-shift mutation c.447delC (p.Ser149fsX156) in exon1 and nonsense mutation c.2034T>G (p.Tyr678X) in exon10, emerged. Neither mutation was detected in control group.

**Conclusions:**

Our results extended the spectrum of *EXT1* mutations, revealing similar incidence of *EXT1* and *EXT2* in Taiwanese MHE patients.

## 1. Introduction

Multiple hereditary exostoses (MHE; also known as multiple osteochondromas, MIM#133700, 133701), most frequent human benign bone tumors, are characterized by multiple outgrowth of bone capped by cartilage, mostly in the metaphyses but also occurring on diaphyses of long bones. Flat bones, vertebrae, and ribs are also affected, skull rarely involved [1]. Onset is variable, from early childhood to puberty, stopping increase until closure of growth plate [2]; prevalence is estimated at 1/50,000 among the European population [1].

MHE may be asymptomatic, but generally clinical presentation is heterogeneous. Because exostoses come from the growth plate, they may consist of deformities and various levels of functional limitation (sensory or motor deficits). Complications, such as compression of nerves and blood vessels, pain caused by pressure on neighboring tissue, and short stature, are also common [3]. Most severe secondary complication is malignant transformation into secondary peripheral chondrosarcoma (in 0.5-5.0% of cases) [4]. Patients with milder forms require no active therapy; physical therapy, pain management, and surgery are common practice in MHE cases, clinical outcome less than beneficial at times [5].

MHE, autosomal dominant disease, links with exostosin 1 (*EXT1*) and 2 (*EXT2*) genes. *EXT1*, assigned to chromosome 8q24.11-q24.13, comprises 11 exons spanning less than 350kb and encoding a polypeptide of 746 amino acids [6, 7]. *EXT2* maps to chromosome 11p11-p11.2, consists of 16 exons, and spans almost 108 kb. Alternative splicing allows three transcript variants of mRNA produced, a major one (transcript variant 2) encoding protein of 718 amino acids [8-10]. Both encode ubiquitously expressed Type-II transmembrane glycoproteins [6, 8, 9] that catalyze elongation of the heparan sulphate- glycosaminoglycan chain of matrix proteoglycans [11].

According to records from the Multiple Osteochondromas Mutation Database (MOdb) (http://medgen.ua.ac.be/LOVDv.2.0/), as well as recent publications, over 440 mutations in *EXT1* and 230 in *EXT2* have been described [12-16]. Depending on race, for all MHE cases, about 56-78% mutation is detected in *EXT1* versus 21-44% for *EXT2* [17]. We sequenced DNA of two Taiwanese patients to determine mutations in *EXT1* or *EXT2* genes, also collecting published genetic analysis of Taiwanese MHE patients, then briefly summarized a mutation profile of the Taiwanese population.

## 2. Patients and Methods

### 2.1. Patients

**Fig. 1:**
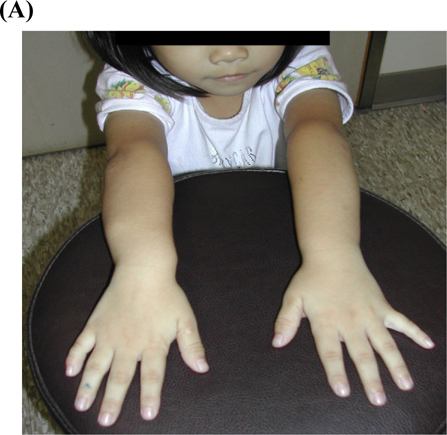

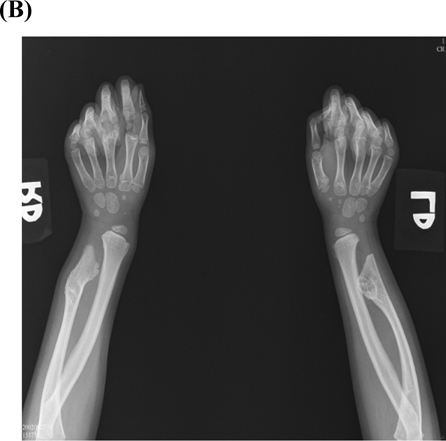
Photograph and radiograph of Patient #1. (A) Both forearms and (B) X-ray image. Note bowing of radius and ulna with bony exostosis over distal ulna.

Patient #1, six-year-old girl, firstborn of nonconsanguineous healthy parents, was brought to our genetic outpatient clinic because of progressive deformation over both forearms for a period of time. Clinical examination revealed shortened forearms with bony projection over distal radial and ulnar sides of right forearm (Figure 1A). Radiologic image disclosed bowing of radius and ulnar bones of both forearms with bony exostosis over distal ulnar bones (Figure 1B).

Patient #2, 23-year-old female, presented typical manifestations of multiple long bones exostosis as index case and was referred by Department of Genetics of National Taiwan University Hospital (Taipei, Taiwan) for further genetic test. Importantly, the propositus’ mother and younger sister and brother had similar clinical phenotypes.

Prior to genetic analysis, informed consent (as per national law) was obtained from adult patients or parents of each study subject.

### 2.2. DNA preparation and sequencing

Genomic DNA extraction from each participant's peripheral blood leukocytes used MagNA Pure LC DNA Isolation Kit (Roche, Mannheim, Germany). Complete *EXT1* and *EXT2* coding regions were amplified according to the protocol published by Plilippe et al. (1997) and Wuyts et al. (1998) [18,19]. In addition, some of the primers were redesigned in this study (Table 1). PCR products were purified from the agarose gel using QIAEX II (Qiagen, Hilden, Germany) and then used for direct sequencing to detect gene mutations. The direct sequencing process was performed using *BigDye* 3.1 Terminator cycle sequencing kit (Applied Biosystems, Forest City, CA) with ABI 3100 Genetic Analyzer (Applied Biosystems, Forest City, CA).

To determine carrier-rate of novel mutations detected in Taiwanese population, *EXT1* gene profile of 100 matched controls was analyzed by procedure mentioned above, reference sequence and base-pair numbers of *EXT1* and *EXT2* obtained from GenBank by accession numbers NM_000127 and NM_207122, respectively.

## 3. Results

**Fig. 2:**
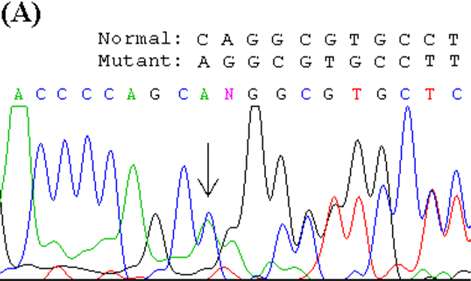

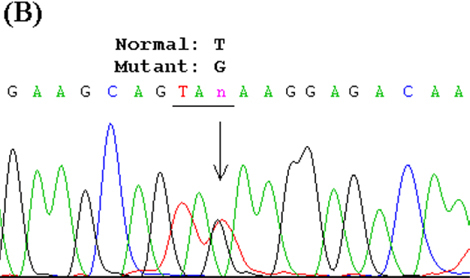
Sequences of novel mutations observed from patients’ EXT1 gene. Arrows indicate mutation sites. (A) A one-base C deleted at nucleotide 447 (c.447delC), (B) G to A substitution at nucleotide 2034 (c.2034T>G) resulting in p.Tyr678X

To identify possible exonic mutations in *EXT1* and *EXT2* causing MHE, entire coding sequence on 26 DNA fragments, each covering an exon and its flanking regions, was amplified. Analysis of *EXT1* and *EXT2* in both patients identified one frame-shift and one nonsense mutation in *EXT1*. To our knowledge, neither has been described in prior article (Figure 2).

In Patient #1, one base C at nucleotide 447 was deleted (c.447delC) in exon1, causing protein translation frame-shift after codon 149, and early terminated at codon 156 (p.Ser149fsX156). This mutation did not appear in her parents’ *EXT1* gene and should be spontaneous mutation. Patient #2 showed nonsense mutation c.2034T>G (p.Tyr678X) located in exon10, inherited from her MHE mother. We detected this mutation site in other family members, confirming this mutation as cosegregated with disease phenotype in those afflicted with MHE. Neither mutation mentioned above was detected in 100 healthy volunteers enrolled in normal control group.

This study found another single polymorphism in *EXT1*, located at nucleotide 1761 (p.Glu587Glu), substitution of G to A. This site was described previously in another population [18].

**Table 1:** Sequences of redesigned primers for *EXT1* and *EXT2* mutation analysis of genomic DNA

Exon	Product size (bp)	PCR primers		Tm
		Forward (5’-3’)	Reverse (5’-3’)	(°C
EXT1-exon1-1	477	GAGAGTTTGAAGTCTTTACAG	TGTGGGTATACGTAGACTTTG	50
EXT1-exon1-2	482	GAAGCGAGATGCCAACTCCAG	CTGTCCTGGGATGATCCTTAG	63
EXT1-exon1-3	458	GACATCGGCCAGGCGATGCTG	GCTCA AAGGGGAAAGAGGACTG	60
EXT1-exon3	330	GCCAGTCATTGAGTTTGTACTG	GACACAGGTAATTTTCTCCTGA	55
EXT1-exon10	261	CTTGTCATCATGTGATAATGGCCC	GAGTGAAGCAAGGGAAGAGGG	56
EXT1-exon11	235	CCTTGCACTTCTCTCATCATTATCC	CCTCAA AGTCGCTCAATGTCTCGG	55
EXT2-exon1b	386	GCCGCGCTTTCAGCATCTT	CCACCTAGTGCCTGGCCCAA	55
EXT2-exon2	670	GTGTCATTTGCCATCCTAAATA	GGCCACTCAAGTATCTCCTG	55
EXT2-exon7b	438	GCCTGGTTGGAGTGAGGCTT	GGCTTGCACCTCCAAGAGCCAT	55

Sequence used refer to GenBank accession numbers NG_007455 (*EXT1*) and NG_007560 (*EXT2*)

## 4. Discussion

*EXT1* and *EXT2* are ubiquitously expressed tumor suppressors of the EXT family, which includes three EXT-like genes (*EXTL1*, *EXTL2*, and *EXTL3*) [20-22]. To date, only mutant *EXT1* and *EXT2* are involved with MHE, no MHE cases have been identified with mutant *EXTL1-3*. All members of the *EXT* gene family share homologous domains: [1] exostosin located in N-terminal region and [2] glycosyltransferase in C-terminal region. The latter is involved in the biosynthesis of heparin sulfate at heparin sulfate proteoglycans [23]. These heparin sulfate proteoglycans play major roles in cell growth/differentiation signal pathways and interact with diffusion of signaling molecules like Indian Hedgehog, an important regulator of chondrocyte proliferation and differentiation in growth plate [24]. These results reflect cartilage growth regulatory function of *EXT1*/ EXT 2 genes [25.26]

Our study found both mutations in *EXT1*. The first was one-base deletion and caused early termination at codon 156 (p.Ser149fsX156), in which neither domain can be translated. Another appeared at c.2034T>G, causing truncated proteins (p.Tyr678X) with glycosyltransferase domain incomplete. Both mutated proteins were in premature form, their structures unstable and soon degraded in the cytoplasm.

*EXT1*/*EXT2* germline mutations have been detected in most MHE cases; *EXT1* mutates more often than *EXT2*, with variable prevalence among populations [17, 27]. Most are frame-shift, nonsense, and splice-site mutations, responsible for premature termination of translation, inducing rapid inactivation and degradation with nearly complete loss of their function [17].

**Fig. 3:**
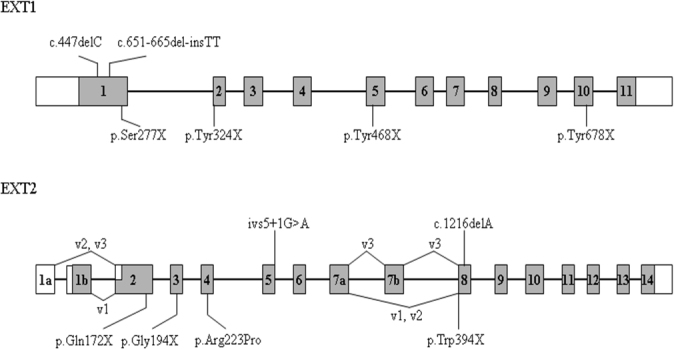
Schematic profile of mutations on *EXT1* and *EXT2* genes in Taiwanese patients with multiple hereditary exostoses. GenBank accession numbers of mRNA sequences: *EXT1*: NM_000127; *EXT2*-v1 (isoform 1): NM_000401, *EXT2*-v2 (isoform 2-Reference): NM_207122, *EXT2*-v3 (isoform 3): NM_001178083.

We collected Taiwanese MHE genetic analysis results from literature and were summary in Figure 3 [28-31]. Total thirteen cases were included, nine were familial cases, and four were sporadic cases (31%), higher than previous study estimated (10%) [17]. Interestingly, in these results, six cases had mutant *EXT1*, seven mutant *EXT2* (54%). This result implies that the incidence of *EXT2* in Taiwanese MHE is equal or slightly high than *EXT1*, which differs from Western populations [12, 17]. According to previous literature, mutations observed in coding region of *EXT1* and *EXT2* that generate frame-shift or nonsense change are dominant [17, 32]. Consider mutation type found in Taiwanese MHE cases: nonsense mutation is major (7/12, 58%), frame-shift type had three (25%), splicing site and missense mutation had one each (8.3%). These proportions concurred with other population studies [12, 13, 15, 17].

To date, we had completed nine MHE genetic studies in Taiwan; all found mutant *EXT1* or *EXT2*. Yet if mutation type is large fragment deletion/duplication, translocation/inversions, or epigenetic variants, it would not be detected by PCR-directed DNA sequencing [33]. According to previous literature, if MHE case could not detect point mutation, the second most probable type is large fragment deletion [12-17]. To augment detection rate, real-time quantitative PCR, multiplex ligation-dependent probe amplification (MLPA), fluorescence *in situ* hybridization (FISH) and DNA microarray can serve for large fragment deletion/duplication mutation analysis [12,13]. If other candidate genes (*EXTL*-1-3) were considered with MHE, linkage analysis should be done first to narrow down the possible gene location.

In conclusion, we identified two novel mutations in *EXT1* from two MHE probands of unrelated Taiwanese families and extended the mutation *EXT1* spectrum. Our patients showed similar incidence of *EXT1* and *EXT2*, lower *EXT1* than among Westerners. Collection mutation data in both genes could help diagnosis and genetic counseling for MHE patients and their families

## Acknowledgements

Study was funded by grant DMR101-095 from China Medical University Hospital.
